# Acetylcholine receptor antagonists in acute respiratory distress syndrome: much more than muscle relaxants

**DOI:** 10.1186/s13054-018-1979-z

**Published:** 2018-05-22

**Authors:** Diana Jansen, Heder de Vries, Leo M. A. Heunks

**Affiliations:** 1 Department of Anesthesiology, Radboud University Medical Center, Nijmegen, The Netherlands; 2 Department of Intensive Care Medicine, VU University Medical Center Amsterdam, Amsterdam, The Netherlands

**Keywords:** Neuromuscular blockers, Acute respiratory distress syndrome, Direct anti-inflammatory effect, Acetylcholine, Acetylcholine receptors, Lung injury markers, Respiratory muscles, Low tidal volume ventilation, Partial neuromuscular blockade

## Abstract

Acetylcholine receptor antagonists have been shown to improve outcome in patients with severe acute respiratory distress syndrome. However, it is incompletely understood how these agents improve outcome. In the current editorial, we discuss the mechanisms of action of acetylcholine receptor antagonists beyond neuromuscular blockade.

## Main text

Non-depolarizing neuromuscular blockers (NMBs), such as rocuronium and cisatracurium, are frequently used in patients with acute respiratory distress syndrome (ARDS). The Lung-Safe study reported that NMBs were used in 6.8% of mild ARDS and up to 37.8% of severe ARDS patients [1]. Three clinical studies on the use of NMB in ARDS have been conducted by Papazian and colleagues [2–4]. In their largest multicenter randomized controlled trial (ACURASYS study) it was demonstrated that continuous cisatracurium for 48 h reduced 90-day mortality (primary outcome) and improved oxygenation, in particular in patients with PaO_2_/FiO_2_ ratio ≤ 120 mmHg [4]. Today, it is incompletely understood how NMBs improve outcome. Possible mechanisms include reduction of oxygen consumption, decrease in cardiac output and pulmonary blood flow, and direct anti-inflammatory effects of NMBs, but the most intuitive mechanism is by abolishing patient breathing effort and thereby limiting the risk of both alveolar collapse and over-distention [5, 6]. However, it is remarkable that no significant differences were found between groups in tidal volume, PEEP, plateau pressure, and minute ventilation [4]. This suggests that other factors, not directly related to respiratory mechanics, may play a role in the beneficial effects of NMBs.

### Acetylcholine receptors

NMBs exert their action through interaction with the acetylcholine receptor (AChR) in the neuromuscular junction. Two major types of AChRs have been characterized: the metabotropic muscarinic receptors (mAChRs) and the ionotropic nicotinic receptors (nAChRs); both are activated by ACh [7]. The nAChR, a ligand-gated ion channel, is primarily found in the neuromuscular junction where binding with acetylcholine results in inflow of sodium and calcium and outflow of potassium, depolarizing the motor endplate and creating a potential that triggers muscle contraction [8, 9]. In addition, nAChRs are expressed by other tissues and cells, including brain, autonomic ganglia, macrophages, endothelial cells, and epithelial cells [7], explaining their involvement in physiological processes such as addiction, inflammation, and metabolic tonus. The mAChR is a G-protein-coupled receptor comprising five subtypes (M1–5) [7, 10] which are also widely expressed throughout the body. Table 1 shows an overview of the most important types of AChRs with their locations and main function.

**Table 1:** Types of AChR with their locations and main function

Type	Location of expression	Function
nAChR		
Muscle-type	Neuromuscular junction	Muscle contraction, mainly by increased Na^+^ and K^+^ permeability
Neuronal-type	Autonomic ganglia	Activation of autonomic nervous system (sympathetic and parasympathetic), mainly by increased Na^+^and K^+^ permeability
	Hippocampus / cortex	Cognition, modulate the induction of synaptic plasticity, effect on learning and memory formation, i.e., can improve neurovascular coupling
	Midbrain	Reward center and initiation of the nicotine addiction process
	Neuro-endocrineneurons in the hypothalamus	Facilitate the Ca^2+^-dependent release of vasopressin and oxytocin
	Others	Improvement of neurovascular coupling (in neurodegenerative disease and ischemia)
mAChR		
M1	Autonomic ganglia	Mediates slow EPSP in postganglionic nerve
	Exocrine glands	Stimulates secretion
	Central nervous system	Activates slow after-depolarizing potentials in neurons
M2	Heart	Reduce of heart rate, contractile forces of the atrium and conduction velocity in AV node
	Central nervous system	Activates slow after-depolarizing potentials in neurons
M3	Smooth muscles	Vasoconstriction, vasodilatation, bronchoconstriction
	Endocrine and exocrine glands	Stimulate secretion
	Central nervous system	Activates slow after-depolarizing potentials in neurons
	Eye	Lacrimation, miosis and accommodation by contraction of the sphincter papillae and ciliary body
M4	Central nervous system	Activates slow after-depolarizing potentials in neurons
M5	Not well known	-

*EPSP* excitatory postsynaptic potential

### NMBs and inflammation

Given the expression of AChRs in different cells throughout the body, it is likely that NMBs exert effects other than neuromuscular blockade. It has been demonstrated in a rat lung injury model that non-depolarizing NMBs (cisatracurium and pancuronium) protect against the development of ventilator-induced lung injury (VILI) through a direct, dose-dependent anti-inflammatory effect mediated by the nAChRα1 expressed on epithelial, endothelial, and CD14^+^ cells [11]. In patients with early ARDS (*N* = 36), continuous administration of cisatracurium for 48 h attenuated pulmonary inflammation (interleukin (IL)8) and systemic inflammation (IL6, IL8) compared to placebo [3]. Recently, new data published in *Critical Care* by Sottile and colleagues [12] support the anti-inflammatory role of NMBs in patients with ARDS. The authors investigated in a secondary analysis of the ARMA trial [13] the effect of NMBs on surfactant protein D (SP-D) and von Willebrand factor (VWF), biomarkers specific for epithelial and endothelial lung injury, respectively, in addition to markers of systemic inflammation (IL8). In the overall cohort (*N* = 446), the use of NMB was significantly associated with an increase in SP-D, but no effect on VWF or IL8. Interestingly, after adjusting for multiple confounders the use of NMBs was associated with a significant decrease in SP-D, VWF, and IL8, but only in patients with a PaO_2_/FiO_2_ ratio ≤ 120 and ventilated with low tidal volumes. In patients with higher PaO_2_/FiO_2_ ratios, or high tidal volumes, NMBs did not affect SP-D, VWF, or IL8. These data provide evidence that NMBs attenuate endothelial and epithelial injury in selected ARDS patients.

### Clinical impact on respiratory muscles and further research

Clinicians may become somewhat confused by the recent literature regarding the role of disuse in the development of critical illness-associated respiratory muscle weakness. On the one hand, excellent data by Goligher et al. [14] demonstrated that in ventilated ICU patients low diaphragm effort is associated with decreased thickness of the diaphragm muscle. In addition, the development of decreased thickness is associated with adverse outcome, including delayed ventilator weaning. On the other hand, the ACURASYS trial [4] demonstrated that 48 h of NMB (resulting in full diaphragm muscle inactivity) improved outcome, including more ventilator-free days (and no development of muscle weakness) compared to placebo. An intriguing explanation is that the beneficial effects of NMBs are at least partly independent of respiratory muscle pump inactivation, but more the result of modulation of inflammation and injury [3, 11, 12] or even unexplored mechanisms. Of note, we have recently demonstrated in a proof of concept study that partial neuromuscular blockade (low dose rocuronium) controls the mechanical effects of high respiratory drive, resulting in pressures consistent with both lung-protective ventilation and diaphragm-protective ventilation [15, 16]. So we might “*ménager la chèvre et le chou*”.

In conclusion, non-depolarizing NMBs have been used for decades in critical care, but we still do not fully understand their effects beyond muscle paralysis. New mechanisms of action may help us to identify patients that benefit the most from the use of NMBs and help us to select appropriate doses.

## References

[1] Bellani G, Laffey JG, Pham T, Fan E, Brochard L, Esteban A (2016). Epidemiology, patterns of care, and mortality for patients with acute respiratory distress syndrome in intensive care units in 50 countries. JAMA.

[2] Gainnier M, Roch A, Forel JM, Thirion X, Arnal JM, Donati S (2004). Effect of neuromuscular blocking agents on gas exchange in patients presenting with acute respiratory distress syndrome. Crit Care Med.

[3] Forel JM, Roch A, Marin V, Michelet P, Demory D, Blache JL (2006). Neuromuscular blocking agents decrease inflammatory response in patients presenting with acute respiratory distress syndrome. Crit Care Med.

[4] Papazian L, Forel JM, Gacouin A, Penot-Ragon C, Perrin G, Loundou A (2010). Neuromuscular blockers in early acute respiratory distress syndrome. N Engl J Med.

[5] Slutsky AS (2010). Neuromuscular blocking agents in ARDS. N Engl J Med.

[6] Bennett S, Hurford WE (2011). When should sedation or neuromuscular blockade be used during mechanical ventilation?. Resp Care.

[7] Albuquerque EX, Pereira EF, Alkondon M, Rogers SW (2009). Mammalian nicotinic acetylcholine receptors: from structure to function. Physiol Rev.

[8] Murray MJ, DeBlock H, Erstad B, Gray A, Jacobi J, Jordan C (2016). Clinical practice guidelines for sustained neuromuscular blockade in the adult critically ill patient. Crit Care Med.

[9] Hunter JM (1995). New neuromuscular blocking drugs. N Engl J Med.

[10] Eglen RM (2012). Overview of muscarinic receptor subtypes. Handb Exp Pharm.

[11] Fanelli V, Morita Y, Cappello P, Ghazarian M, Sugumar B, Delsedime L (2016). Neuromuscular blocking agent cisatracurium attenuates lung injury by inhibition of nicotinic acetylcholine receptor-alpha1. Anesthesiology.

[12] Sottile G, Albers D, Moss MM. Neuromuscular blockade is associated with the attenuation of biomarkers of epithelial and endothelial injury in patients with moderate-severe ARDS. Crit Care. 2018;22(1):63. 10.1186/s13054-018-1974-4 PMC5845220 29523157

[13] Brower RG, Matthay MA, Morris A, Schoenfeld D, Thompson BT, Acute Respiratory Distress Syndrome Network (2000). Ventilation with lower tidal volumes as compared with traditional tidal volumes for acute lung injury and the acute respiratory distress syndrome. N Engl J Med.

[14] Goligher EC, Dres M, Fan E, Rubenfeld GD, Scales DC, Herridge MS (2018). Mechanical ventilation-induced diaphragm atrophy strongly impacts clinical outcomes. Am J Respir Crit Care Med.

[15] Doorduin J, Nollet JL, Roesthuis LH, van Hees HW, Brochard LJ, Sinderby CA (2017). Partial neuromuscular blockade during partial ventilatory support in sedated patients with high tidal volumes. Am J Respir Crit Care Med.

[16] Heunks L, Ottenheijm C (2018). Diaphragm-protective mechanical ventilation to improve outcomes in ICU patients?. Am J Respir Crit Care Med.

